# The association between Albumin-Corrected Anion Gap (ACAG) and the risk of acute kidney injury in patients with acute pancreatitis: A retrospective analysis based on the MIMIC-IV database

**DOI:** 10.1371/journal.pone.0330458

**Published:** 2025-08-22

**Authors:** Jiaxiang Bian, Xiaoyang Wang, Youli Chen, Guiyang Lu, Luanluan Zhang, Xiaoyan Tu, Shuling Wang, Weibin Huang, Cunrong Chen

**Affiliations:** 1 Department of Critical Care Medicine, Fujian Medical University Union Hospital, Fuzhou, Fujian, China; 2 Department of Critical Care Medicine, Mengchao Hepatobiliary Hospital of Fujian Medical University, Fuzhou Fujian, China; 3 Department of Critical Care Medicine, Quanzhou First Hospital Affiliated to Fujian Medical University, Quanzhou Fujian, China; 4 Department of Intensive Care Medicine, the First Affiliated Hospital of Xiamen University, Xiamen Fujian, China; 5 Department of Emergency Intensive Care Unit, The First Affiliated Hospital of Zhengzhou University, Zhengzhou Henan, China; University of Rijeka Faculty of Medicine: Sveuciliste u Rijeci Medicinski fakultet, CROATIA

## Abstract

**Purpose:**

Acute kidney injury (AKI), a common and severe complication of acute pancreatitis (AP), is significantly linked to patient prognosis. Albumin-corrected anion gap (ACAG) is a modified acid-base balance assessment metric with potential clinical significance in various critical illnesses. However, the role of ACAG in forecasting the risk of AKI in AP patients remains unclear. This study sheds light on the relationship between ACAG levels and AKI risk in the AP population.

**Methods:**

This retrospective study utilized data from the MIMIC-IV database, including 1,552 adult patients diagnosed with AP during their stay in the intensive care unit (ICU). ACAG was calculated using a standard formula, and patients were grouped according to their ACAG levels. Cox proportional hazards and restricted cubic spline (RCS) models were employed to assess the correlation of ACAG levels with AKI risk in AP patients. The incidence of AKI was the primary outcome, and in-hospital mortality was the secondary outcome. Differences in primary and secondary outcomes between ACAG groups were evaluated through Kaplan-Meier (KM) survival analysis. Subgroup analyses were performed for examining the influence of confounding factors.

**Results:**

Higher ACAG levels were significantly related to an elevated risk of AKI. The RCS model demonstrated a nonlinear correlation between higher ACAG levels and increased AKI risk in the AP cohort, and a linear association of ACAG with in-hospital death. KM survival analysis showed that patients exhibiting higher ACAG levels had poorer renal function outcomes and higher ICU mortality. Subgroup analyses further proved this correlation across varied patient characteristics.

**Conclusions:**

Elevated ACAG is an independent predictor of AKI risk in the AP cohort. ACAG may be useful for early AKI risk stratification and clinical decision-making in critically ill AP sufferers.

## 1. Introduction

Acute pancreatitis (AP) is a prevalent acute inflammatory abdominal illness with a wide spectrum of severity. It can manifest as mild pancreatitis or progress to severe conditions like systemic inflammatory response syndrome (SIRS) and multiple organ dysfunction syndrome (MODS) [1,2]. Among AP patients, approximately 15–20% have severe acute pancreatitis (SAP), which is a severe condition linked to single or multiple organ failures and local or systemic complications, with a death rate of 36% − 50%. [3–6] Acute kidney injury (AKI), a common and serious complication in AP patients, significantly increases both mortality and healthcare burdens [7,8]. Furthermore, AKI is independently related to higher mortality rates in AP sufferers [9]. Therefore, early identification and risk prediction of AKI in AP patients in the intensive care unit (ICU) hold crucial clinical significance.

Albumin-corrected anion gap (ACAG) is an adjusted anion gap calculated after correcting for serum albumin levels, offering a more accurate assessment method of acid-base balance [10] Traditional anion gap (AG) may lead to misjudgments in acid-base imbalance due to its failure to account for the effects of hypoalbuminemia [11]. In contrast, ACAG, by adjusting for albumin concentration, more accurately reflects the underlying metabolic acidosis status of the body. ACAG is closely correlated with the prognosis of various critical diseases [12–15]. Nevertheless, the link of ACAG to AKI in AP patients is not elucidated.

The MIMIC-IV database is an open-access resource containing extensive clinical data and laboratory indicators from ICU patients, providing a solid data foundation for analyzing the connection of ACAG with AKI in AP patients. This study aims to utilize the MIMIC-IV database to systematically investigate the association between ACAG and AKI in the AP population and further examine the potential clinical value of ACAG in forecasting the risk of AKI. The ultimate goal is to offer a new reference for the early management of critically ill AP sufferers.

## 2. Methods

### 2.1. Data source

The raw data were sourced from the Medical Information Mart for Intensive Care-IV (MIMIC-IV) database (https://mimic.mit.edu). This database contains detailed clinical data from ICU patients of an academic medical center in the United States between 2008 and 2019. It includes demographic information, laboratory test results, medication records, and prognostic outcomes [16]. To gain access to the database, the author, Jiaxiang Bian, completed the Collaborative Institutional Training Initiative (CITI) course (Record ID: 13501033) and obtained necessary permissions for using the database. The MIMIC-IV project was approved by the Institutional Review Boards of the Massachusetts Institute of Technology (Cambridge, MA, USA) and the Beth Israel Deaconess Medical Center. Patient information has been anonymized, and thus informed consent was not required.

### 2.2. Study population

**Fig 1:**
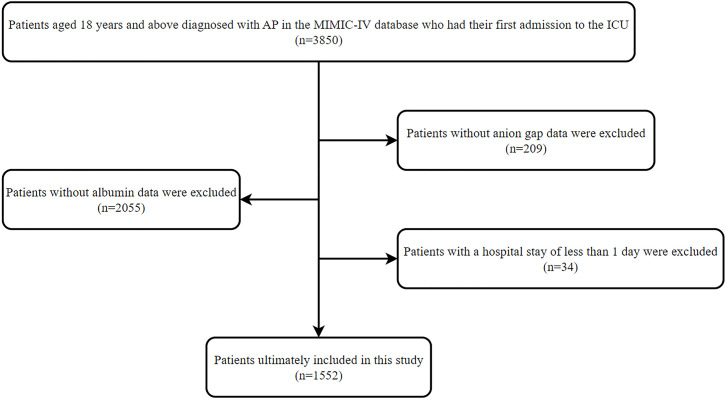
Flowchart of study population selection.

This study focused on a cohort comprising 3,850 patients who were diagnosed with AP and had non-continuous ICU admissions (aged ≥18) from MIMIC-IV. The diagnosis of AP was confirmed in adherence to the International Classification of Diseases, 9th edition (ICD-9) and 10th edition (ICD-10) codes. Only the first hospital admission for each patient was considered if they had multiple admissions. To ensure data completeness, patients with missing albumin or AG values, as well as those who stayed fewer than 24 hours in hospital, were removed. At last, 1,552 patients were eligible for the analysis. The specific patient selection is presented in Fig 1.

### 2.3. Study variables

This study utilized Structured Query Language (SQL) and PostgreSQL (version 14.2) to extract baseline characteristics from MIMIC-IV, including demographic information, laboratory parameters, comorbidities, incidence of AKI, and in-hospital mortality. Demographic data encompassed gender, age, race, marital status, and body mass index (BMI). Vital signs encompassed respiratory rate (RR), heart rate (HR), systolic blood pressure (SBP), and diastolic blood pressure (DBP). Laboratory parameters included red blood cells (RBC), hemoglobin (Hb), white blood cells (WBC), platelets (PLT), pO₂, pCO₂, pH, HCO₃ ⁻ , sodium, potassium, calcium, magnesium, lactate, glucose, triglycerides (TG), alanine aminotransferase (ALT), aspartate aminotransferase (AST), total bilirubin (TBIL), serum creatinine (SCr), urinary creatinine (UCr), blood urea nitrogen (BUN), AG, and albumin (ALB). Comorbidities and personal medical history determined according to ICD-9 and ICD-10 codes, were hypertension, diabetes mellitus (DM), hyperlipidemia, coronary heart disease (CHD), heart failure (HF), atrial fibrillation (AF), chronic obstructive pulmonary disease (COPD), cirrhosis, chronic pancreatitis (CP), pancreatic cysts and pseudocysts (PC and PPC), chronic kidney disease (CKD), sepsis, and shock. Sequential Organ Failure Assessment (SOFA) score and SIRS score were employed for evaluating the severity of patients after admission. Surgical history included resection of the gallbladder and common bile duct dilation. In addition, to explore whether albumin infusion as an interventional measure might influence outcomes, this variable was incorporated into the analysis.

The exposure variable was ACAG calculated as follows: AG (mmol/l) = (sodium + potassium) − (chloride + bicarbonate) [17]; ACAG = AG + {4.4-[ALB(g/dl)]} × 2.5 [10]. The primary outcome was defined as the occurrence of AKI from ICU admission to hospital discharge. AKI was defined according to the Kidney Disease: Improving Global Outcomes (KDIGO) guidelines as an increase in serum creatinine (SCr) to ≥1.5 times the baseline value within the prior 7 days, an absolute increase in SCr of ≥0.3 mg/dL within 48 hours, or a reduction in urine output to <0.5 mL/kg/h for at least 6 hours [18]. The secondary outcome was the in-hospital death rate of AP sufferers.

### 2.4. Statistical analysis

All statistical analyses were conducted via R 4.4.2. For repeatedly measured laboratory parameters, the first recorded value within 3 days of admission was used. During the data preprocessing stage, timestamps were handled using the as. POSIXctfunction in R. Data entries were subsequently categorized based on the time interval (i.e., AKI onset time minus the parameter measurement time), with the data classified as occurring either before or after the AKI event. To reduce reverse causality bias, data collected after the onset of AKI events were considered invalid. Given the common occurrence of missing laboratory values in MIMIC-IV, the proportions of missing data for each continuous variable were calculated with reference to previous studies [19,20] (S1 Table). For variables with a missing value proportion of less than 20%, missing values were predicted and imputed using a random forest-based multiple imputation method (missForest R). The tbl_summary function was used to compare data before and after multiple imputation (S2 Table). For variables with missing value proportions exceeding 20%, the missing part was treated as a separate category during the analysis.

For continuous variables in normal distribution, means and standard deviations were used, and differences between groups were compared via independent sample t-tests. For continuous variables not in a normal distribution, medians and interquartile ranges (IQR) were used for description, and group differences were compared through non-parametric tests (Mann-Whitney U test). For categorical variables, frequencies and percentages were used, and between-group comparisons were conducted through chi-squared or Fisher’s exact tests.

Kaplan-Meier (KM) survival curves were used to estimate the cumulative probabilities of AKI incidence and in-hospital survival across different ACAG level groups, and the differences between groups were assessed through the log-rank test.

To explore the nonlinear association of ACAG with AKI and in-hospital mortality, restricted cubic splines (RCS) were constructed. A piecewise fitting approach was used to describe the continuous relationship between ACAG and outcome variables, and smoothed curves were plotted to visually present the dose-response relationship. The study employed four default knots located at the 5th, 35th, 65th, and 95th percentiles, as recommended by Harrell et al. [21] to balance model fitting with robustness.

The independent correlations between ACAG and the incidence of AKI and in-hospital death among AP patients were investigated through Cox proportional hazards models. Confounding factors were progressively adjusted for as follows: Model 1 has no adjustment for confounding factors; In Model 2, demographic information like age, gender, race, and marital status were adjusted; In addition to the adjustments in Model 2, further adjustment for clinical variables, including BMI, sodium, potassium, calcium, magnesium, bicarbonate, ALT, AST, SCr, RBC, Hb, WBC, PLT, hypertension, DM, hyperlipidemia, CHD, COPD, CP, CKD, shock and albumin infusion was made in Model 3. To avoid multicollinearity, variance inflation factors (VIFs) were calculated for each variable when constructing multivariable models (Models 2 and 3), and variables with a VIF greater than 5 were excluded.

Stratified analyses were conducted based on key characteristics (e.g., age, gender, race, BMI, hypertension, DM, hyperlipidemia, CHD, COPD, CKD, CP, pancreatic cysts and pseudocysts, HF, AF, liver cirrhosis, and sepsis) to explore whether the effect of ACAG on outcome variables was consistent across subgroups. To elucidate the modifying effects of specific variables on the link between ACAG to outcomes, interaction tests were carried out. P < 0.05 denoted statistical significance.

## 3. Results

### 3.1 Population and baseline characteristics

Our study encompassed 1,552 patients, among whom 350 (23%) developed AKI, and 75 (4.8%) experienced in-hospital mortality. Within the entire cohort, a relatively higher proportion of patients were male (53%), 84% of the patients were over 40 years old, and 70% were of Caucasian descent.

S3 Table shows the baseline characteristics of patients stratified into four quartiles according to their ACAG levels (Q1: 8.50–14.69, Q2: 14.69–16.75, Q3: 16.75–19.25, Q4: 19.25–55.00). As ACAG levels increased, the incidence of AKI significantly escalated (9.3% vs. 17% vs. 25% vs. 40%, P < 0.001). Furthermore, the in-hospital death rate was notably higher in the Q4 group, with a significantly greater proportion of patients suffering from diabetes mellitus (DM), hyperlipidemia, HF, AF, COPD, CKD, and sepsis. Regarding clinical and laboratory parameters, the Q4 group exhibited significant increases in length of stay, SOFA score, HR, WBC, glucose (Glu), ALT, AST, TBIL, SCr, urine creatinine (UCr), BUN, and AG, while bicarbonate (HCO₃⁻), calcium (Ca), and ALB levels were significantly lower.

S4 Table summarizes the baseline characteristics of AKI and non-AKI cohorts. Compared with the non-AKI group, the AKI group exhibited a significantly elevated in-hospital mortality rate, and a notably greater proportion of patients with DM, hyperlipidemia, CHD, HF, AF, COPD, liver cirrhosis, pneumonia (PC and PPC), CKD, and sepsis. Clinically, the AKI group showed significantly elevated length of stay, SOFA score, SIRS score, WBC, potassium (K), glucose (Glu), AST, TBIL, SCr, UCr, BUN, and AG, while HCO₃ ⁻ , Ca, and Alb levels were significantly reduced.

### 3.2 Survival analysis

**Fig 2:**
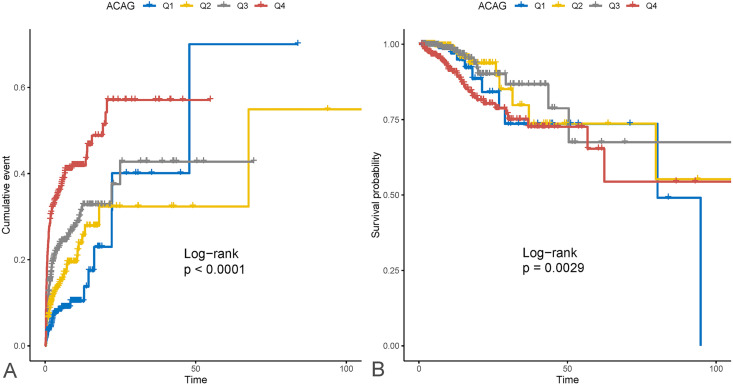
KM analysis results. **(A)** AKI event occurrence curve; **(B)** Survival curve for AP patients.

KM survival curves indicated notable variances in the cumulative incidence of AKI and in-hospital survival rates between the groups stratified by ACAG levels (AKI incidence: Log-rank test, P < 0.0001; in-hospital survival rate: Log-rank test, P = 0.0029). Patients with higher ACAG levels exhibited a higher risk of AKI, along with a significantly lower in-hospital survival rate. The detailed findings are presented in Fig 2.

### 3.3 RCS analysis

**Fig 3:**
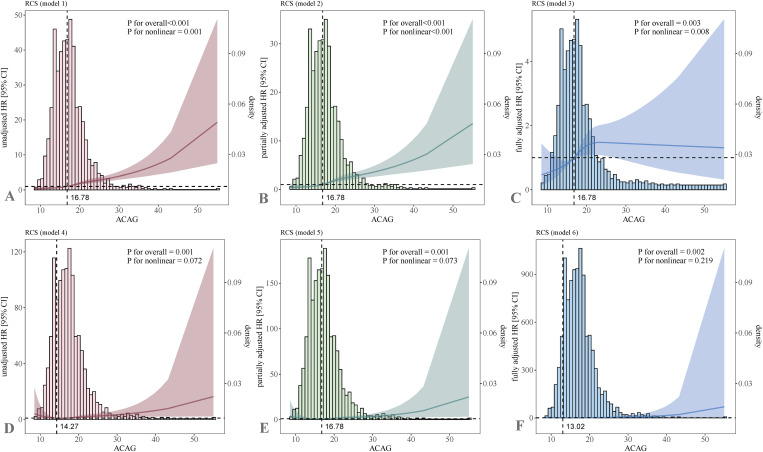
Association between ACAG and AKI risk and in-hospital mortality in AP patients. **(A-C)** AKI incidence; **(D-F)** in-hospital mortality.

RCS analysis revealed a non-linear association between ACAG and AKI in the AP cohort in the unadjusted model (P-overall < 0.001, P-nonlinear = 0.001), partially adjusted model (P-overall < 0.001, P-nonlinear < 0.001), and fully adjusted model (P-overall = 0.003, P-nonlinear = 0.008). Detailed results can be found in Figs 3A–C. Furthermore, a linear correlation of ACAG with inpatient death was observed in AP sufferers in the unadjusted model(P-overall = 0.001, P-nonlinear = 0.072), partially adjusted model (P-overall = 0.001, P-nonlinear = 0.073), and the fully adjusted model (P-overall = 0.002, P-nonlinear = 0.219). Detailed results can be found in Figs 3D–F. As ACAG levels increased, the risk of AKI exhibited an exponential growth trend, with a more pronounced increase beyond a certain threshold (e.g., ACAG > 16.78 mmol/L). In contrast, in-hospital mortality also increased with rising ACAG levels, although the curve was more gradual.

### 3.4. Cox proportional hazards models

The results of Cox proportional hazards models indicated ACAG as an independent predictor of both AKI incidence and in-hospital mortality. When ACAG was included as a continuous variable in the Cox proportional hazards models, a statistically significant association between ACAG and AKI was demonstrated across unadjusted, partially adjusted, and fully adjusted models. As ACAG levels increased, both AKI risk and in-hospital mortality significantly elevated. Specifically, in the unadjusted model 1, each 1 mmol/L increase in ACAG was linked to a significantly higher risk of AKI (HR = 1.108, 95% CI: 1.090–1.126, P < 0.001), as well as a significantly increased risk of in-hospital death (HR = 1.070, 95% CI: 1.029–1.113, P < 0.001).

When ACAG was included as a categorical variable in the model, the results showed that patients in the high ACAG group had a significantly higher AKI risk than the low ACAG cohort. This association remained significant even after all confounding factors were adjusted, validating the strong correlation between high ACAG levels and adverse outcomes. Detailed information is presented in Table 1.

**Table 1:** The results of Cox proportional hazards models.

	Model 1			Model 2			Model 3		
Characteristic	HR	95% CI	p-value	HR	95% CI	p-value	HR	95% CI	p-value
Association between ACAG and AKI									
ACAG (per one unit)	1.108	1.090, 1.126	<0.001	1.099	1.081, 1.118	<0.001	1.032	1.002, 1.064	0.035
ACAG^a^ (quartiles)	p of trend: < 0.001			p of trend: < 0.001			p of trend: < 0.001		
Q1	—	—		—	—		—	—	
Q2	1.762	1.176, 2.639	0.006	1.671	1.112, 2.512	0.014	1.479	0.975, 2.246	0.066
Q3	2.678	1.828, 3.922	<0.001	2.583	1.758, 3.796	<0.001	1.870	1.245, 2.809	0.003
Q4	4.712	3.271, 6.787	<0.001	4.359	3.013, 6.305	<0.001	2.186	1.408, 3.396	<0.001
Association between ACAG and In-hospital mortality									
ACAG (per one unit)	1.070	1.029, 1.113	<0.001	1.072	1.028, 1.118	0.001	1.115	1.048, 1.186	<0.001
ACAG^a^ (quartiles)	p of trend: 0.135			p of trend: 0.359			p of trend: 0.122		
Q1	—	—		—	—		—	—	
Q2	0.653	0.277, 1.542	0.331	0.537	0.226, 1.276	0.159	0.489	0.186, 1.290	0.148
Q3	0.680	0.299, 1.548	0.358	0.567	0.246, 1.305	0.182	0.786	0.302, 2.044	0.621
Q4	1.721	0.879, 3.368	0.113	1.396	0.700, 2.785	0.343	1.855	0.759, 4.536	0.176

Model 1 was unadjusted

Model 2 was adjusted for gender, age, race and marital status.

Model 3 was adjusted for the variables in model 2 and further adjusted for BMI, Na, K, Ca, Mg, HCO₃ ⁻ , ALT, AST, SCr, RBC, Hb, WBC, PLT, hypertension, DM, hyperlipidemia, CHD, COPD, CP, CKD, shock, and albumin infusion.

^a^ACAG index: Q1: 8.50 ~ 14.69, Q2: 14.69 ~ 16.75, Q3: 16.75 ~ 19.25, Q4: 19.25 ~ 55.00;

### 3.5 Subgroup analysis and interaction test

**Fig 4:**
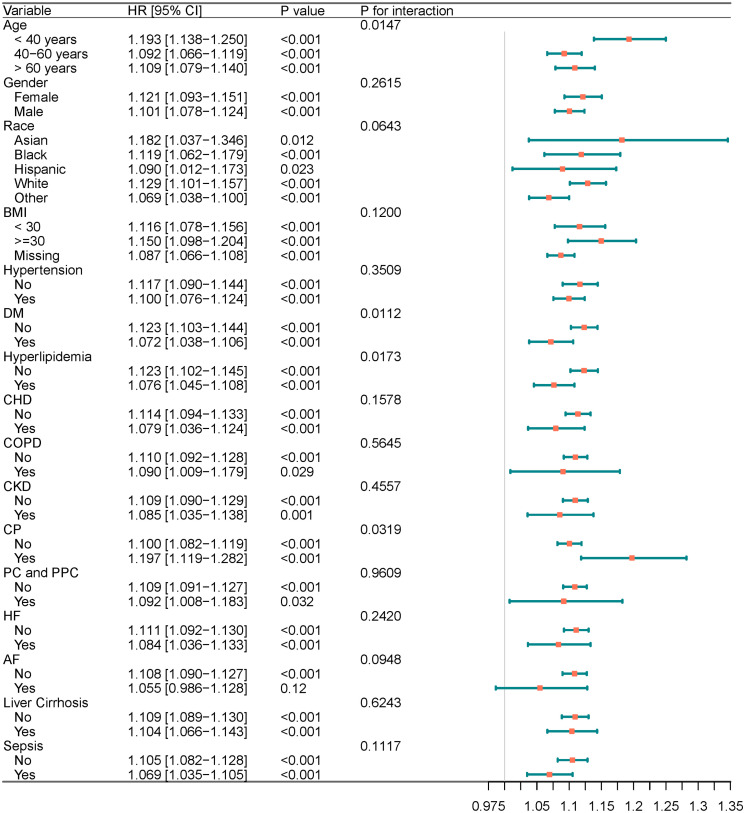
Forest plots of hazard ratios for the primary endpoint in different subgroups. Notes:HRs are per 1-unit increase in ACAG.

Subgroup analysis revealed that the association between ACAG and AKI risk was consistent across most subgroups (e.g., gender, race, BMI, hypertension, CHD, COPD, CKD, post-cardiac surgery, postoperative pneumonia, HF, AF, liver cirrhosis, and sepsis) (P for interaction > 0.05). However, interaction testing indicated a significant interaction effect between age, diabetes mellitus, hyperlipidemia, and ACAG (P for interaction < 0.05), suggesting that these factors may influence the strength of the effect of ACAG on outcomes. Detailed results are presented in Fig 4.

## 4. Discussion

AKI is considered a major cause of death in AP sufferers in ICU. Plenty of studies reported a wide range of AKI incidence in AP patients, from 7.9% to 69.3%, a disparity that may be attributed to variations in the severity of the conditions of the included patients [7,9,22–26]**.** In this study, AKI was observed in 23% of AP patients, a result consistent with previous findings. Research has shown that AKI increases the mortality rate in AP patients by approximately fivefold [23]. In our study, the in-hospital death rate in the AKI population was significantly higher than that in the non-AKI cohort(18% vs. 1.1%). Therefore, early detection of high-risk AP patients for AKI and timely intervention are essential in lowering the incidence of AKI and ameliorating outcomes.

AP patients, especially those with SAP, frequently experience disturbances in acid-base homeostasis during disease progression. Experimental studies have previously demonstrated that metabolic acidosis may exacerbate experimental AKI [27]. However, the exact mechanisms underlying the aggravation of AKI by metabolic acidosis remain to be fully elucidated. Bugarski et al. suggested that metabolic acidosis may contribute to AKI by altering NAD and lipid metabolism in proximal renal tubules [28]. As the kidney is a critical organ in maintaining systemic acid-base balance, the development of AKI inevitably disrupts this homeostasis, thereby creating a vicious cycle that further aggravates renal injury [29].

Serum AG, a reliable and easily accessible clinical marker, can be used to determine the type of metabolic acidosis and evaluate the acid-base status [30]. AG correlates with the mortality and length of ICU stay [31–36], and it is widely employed to assess the prognosis of many illnesses like acute myocardial infarction [37], HF [38], influenza [39], acute pesticide intoxication [40], and disseminated intravascular coagulation [41]. However, AG is susceptible to the concentration of ALB, and in cases of hypoalbuminemia, AG may be underestimated [10,42,43]. Specifically, for every 1 g/dL change in serum albumin, a corresponding 2.3 to 2.5 mmol/L change in the serum AG was observed [17]. In our study, the ALB level was much lower in individuals with AKI than in those without AKI, suggesting that the direct use of AG may have limitations under these conditions. The introduction of ACAG can effectively overcome this issue and more accurately reflect the patient’s true metabolic state.

Our findings proved that ACAG was a crucial independent predictor of both AKI and in-hospital mortality. The risk of adverse outcomes significantly increased as ACAG levels rose. Cox proportional hazards models further confirmed this association and demonstrated that even after confounding factors were adjusted, elevated ACAG levels remained an independent risk factor for AKI and in-hospital mortality. Moreover, RCS analysis revealed a nonlinear correlation of ACAG with outcomes, particularly at high ACAG levels, where the risks of AKI and mortality increased at an accelerated rate. This finding suggests that high ACAG may reflect more severe metabolic disturbances or latent pathophysiological abnormalities, with its threshold effect providing an important basis for early clinical intervention.

Notably, ACAG has been proven to be an independent risk factor for mortality, although its threshold varies slightly across different studies. For example, in patients with AP, the critical value of ACAG is 19.03 mmol/L [44], while for severe AKI patients, it is 20 mmol/L [45]; in the critically ill AKI cohort undergoing continuous renal replacement therapy (CRRT), it is 20 mmol/L [46]; for those with acute myocardial infarction [47] and cardiac arrest [48], it is also 20 mmol/L; for trauma patients, it is 20.375 mmol/L [14]; for asthma patients, it is 20.38 mmol/L [49]; and for ICU patients with sepsis, it is 21.25 mmol/L [50]. In this study, ACAG was categorized into four intervals based on median and IQR. The incidence of AKI in AP sufferers significantly increased in the interval of >19.25 mmol/L. Furthermore, the RCS plot demonstrated a marked increase in AKI risk in AP patients once ACAG levels exceeded 16.78 mmol/L. Nonetheless, the findings suggest that clinicians focus more on patients who have higher ACAG values.

In the subgroup analysis, this study revealed that although the overall association between ACAG and outcomes remained consistent across most subgroups, significant interactions between ACAG and factors such as age, diabetes mellitus (DM), hyperlipidemia, and CP were observed. This suggests that these variables might modulate the predictive effectiveness of ACAG for AKI risk in AP patients. Previous research has shown that age influences the composition of blood acid-base balance, and with increasing age, the severity of metabolic acidosis may be exacerbated due to declining renal function [51]. Among patients with diabetes, chronic hyperglycemia, insulin resistance, impaired lactate and ketone metabolism, and the effects of various antidiabetic medications may all influence metabolic acidosis and the AG [52–58]. Moreover, diabetes itself can lead to varying degrees of renal impairment [59,60]. In patients with hyperlipidemia, elevated circulating lipid levels may interfere with ion measurements in laboratory assays, potentially leading to abnormal AG results [61–63]. Existing pancreatic dysfunction in CP patients may worsen the acid-base imbalance [64]. Moreover, although racial differences did not exhibit significant interaction effects in subgroup analyses, prior studies have reported a higher prevalence of metabolic acidosis among CKD patients who are Asian, Black, or Hispanic compared to non-Hispanic Whites [65]. Variations in acid-base metabolism across racial groups may be attributable to differences in dietary habits and genetic predispositions [66]. These findings underscore the importance of precise assessment and individualized management for specific high-risk patient groups. Future studies could focus on patient populations with differing comorbidities to further clarify the impact of ACAG on AKI occurrence and prognosis in patients with AP.

Our study also has some limitations. First, due to its retrospective nature, causal relationships cannot be definitively established, and only associations between variables can be explored. Additionally, several unmeasured confounding factors, such as fluid resuscitation practices and medication usage, may have influenced the results. Second, the exclusion of cases with uncomputable ACAG values may have introduced residual selection bias. Moreover, certain laboratory indicators were missing, and although multiple imputation methods were employed to address this, potential biases remain difficult to fully eliminate. Additionally, the study sample was predominantly drawn from a single-center public database with a majority of White patients, which may lead to selection bias and limit the applicability of our conclusions to other populations, such as those in Africa or Asia.

Our results unveil that ACAG, as a readily accessible laboratory marker, can effectively forecast AKI risk and in-hospital mortality in the AP cohort. Future multicenter prospective cohort studies are warranted to validate the predictive value of ACAG across diverse populations and explore whether interventions aimed at reducing ACAG levels (e.g., albumin infusion, management of acidosis) may improve clinical outcomes. Furthermore, existing medical databases can be further leveraged for in-depth data mining, in conjunction with machine learning and other advanced analytical techniques [67,68] to further optimize prediction models of ACAG combined with other clinical variables, thereby enhancing the accuracy and efficacy of risk assessment.

## 5. Conclusion

This study confirms that ACAG is a significant prognostic indicator for the AP population, and elevated levels significantly raise the risk of AKI and in-hospital mortality. Further research may pave the way for the integration of ACAG into standardized clinical risk assessment tools and provide a more scientific basis for personalized treatment in AP patients.

## Supporting information

S1 Table Proportion of missing values for each variable. (DOCX)

S2 Table Sensitivity analysis (comparison of data before and after missing value interpolation). (DOCX)

S3 Table The baseline characteristics of patients (According to ACAG levels). (DOCX)

S4 Table The baseline characteristics of patients (According to AKI and non-AKI cohorts). (DOCX)
