# Supplementary material for: The association between Albumin-Corrected Anion Gap (ACAG) and the risk of acute kidney injury in patients with acute pancreatitis: A retrospective analysis based on the MIMIC-IV database
Source: PLoS One. 2025 Aug 22;20(8):e0330458. doi: 10.1371/journal.pone.0330458 (PMC12373200; doi:10.1371/journal.pone.0330458)
Supplement: S1 Table — (DOCX) [file pone.0330458.s001.docx]

**Supplementary appendix**

**Supplementary Table 1.** Proportion of missing values for each variable

| Variables | Missing Rate (%) |
| --- | --- |
| BMI (kg/m2) | 35.6 |
| SOFA score | 67.1 |
| SIRS score | 67.1 |
| RR (insp/min) | 68.6 |
| HR (bpm) | 68.6 |
| SBP (mmHg) | 39.4 |
| DBP (mmHg) | 39.4 |
| RBC (m/uL) | 0.3 |
| Hb (g/dL) | 0.5 |
| WBC (K/uL) | 0.4 |
| PLT (K/uL) | 0.4 |
| pO2 (mmHg) | 74.4 |
| pCO2 (mmHg) | 74.4 |
| PH | 71.6 |
| HCO3 (mEq/L) | 0.1 |
| Na (mEq/L) | 0 |
| K (mEq/L) | 0 |
| Ca (mg/dL) | 2.1 |
| Mg (mg/dL) | 1.9 |
| Lactate (mmol/L) | 65.1 |
| Glu (mg/dL) | 0.1 |
| TG (mg/dL) | 66.6 |
| ALT (IU/L) | 2.3 |
| AST (IU/L) | 1.9 |
| TBIL (mg/dL) | 2.3 |
| SCr (mg/dL) | 0 |
| UCr (mg/dL) | 0.3 |
| BUN (mg/dL) | 0.3 |
| AG (mEq/L) | 0 |
| Alb (g/dL) | 0 |
| ACAG | 0 |

Abbreviation: BMI (Body Mass Index), SOFA (Sequential Organ Failure Assessment), SIRS (Systemic Inflammatory Response Syndrome), RR (Respiratory Rate), HR (Heart Rate), SBP (Systolic Blood Pressure), DBP (Diastolic Blood Pressure), RBC (Red Blood Cell), Hb (Hemoglobin), WBC (White Blood Cell), PLT (Platelets), TG (Triglyceride), ALT (Alanine Aminotransferase), AST (Aspartate Aminotransferase), TBIL (Total Bilirubin), SCr (Serum Creatinine), UCr (Urine Creatinine), BUN (Blood Urea Nitrogen), AG (Anion Gap), Alb (Albumin), ACAG (Albumin Corrected Anion Gap)
