# Supplementary material for: The association between Albumin-Corrected Anion Gap (ACAG) and the risk of acute kidney injury in patients with acute pancreatitis: A retrospective analysis based on the MIMIC-IV database
Source: PLoS One. 2025 Aug 22;20(8):e0330458. doi: 10.1371/journal.pone.0330458 (PMC12373200; doi:10.1371/journal.pone.0330458)
Supplement: S2 Table — (DOCX) [file pone.0330458.s002.docx]

**Supplementary appendix**

**Supplementary Table 2.** Sensitivity analysis (comparison of data before and after missing value interpolation)

| **Characteristic** | **After Imputation** N = 1,552*^1^* | **Before Imputation** N = 1,552*^1^* | **p-value***^2^* |
| --- | --- | --- | --- |
| RBC (m/uL) | 3.79 [3.36-4.25] | 3.79 [3.36-4.26] | 0.987 |
| Hb (g/dL) | 11.57 ± 2.03 | 11.57 ± 2.04 | 0.996 |
| WBC (K/uL) | 9.0 [6.2-13.1] | 9.0 [6.2-13.1] | 0.983 |
| PLT (K/uL) | 208 [147-275] | 208 [147-275] | 0.966 |
| HCO₃⁻ (mEq/L) | 24.0 [21.0-26.0] | 24.0 [21.0-26.0] | 0.979 |
| Ca (mg/dL) | 8.50 [7.90-8.90] | 8.40 [7.90-8.90] | 0.872 |
| Mg (mg/dL) | 1.87 [1.70-2.10] | 1.90 [1.70-2.10] | 0.982 |
| Glu (mg/dL) | 107 [89-140] | 107 [89-140] | 0.976 |
| ALT (IU/L) | 52 [22-163] | 51 [22-153] | 0.667 |
| AST (IU/L) | 59 [26-142] | 57 [26-135] | 0.610 |
| TBIL (mg/dL) | 0.90 [0.50-2.49] | 0.90 [0.50-2.30] | 0.571 |
| UCr (mg/dL) | 13 [9-23] | 13 [9-23] | 0.959 |
| BUN (mg/dL) | 13 [9-23] | 13 [9-23] | 0.961 |
| *^1^* Median [Q1-Q3]; Mean ± SD | | | |
| *^2^* Wilcoxon rank sum test | | | |

Abbreviation: RBC (Red Blood Cell), Hb (Hemoglobin), WBC (White Blood Cell), PLT (Platelets), ALT (Alanine Aminotransferase), AST (Aspartate Aminotransferase), TBIL (Total Bilirubin), UCr (Urine Creatinine), BUN (Blood Urea Nitrogen)
