# Supplementary material for: The association between Albumin-Corrected Anion Gap (ACAG) and the risk of acute kidney injury in patients with acute pancreatitis: A retrospective analysis based on the MIMIC-IV database
Source: PLoS One. 2025 Aug 22;20(8):e0330458. doi: 10.1371/journal.pone.0330458 (PMC12373200; doi:10.1371/journal.pone.0330458)
Supplement: S4 Table — (DOCX) [file pone.0330458.s004.docx]

**Supplementary appendix**

**Supplementary Table 4.** The baseline characteristics of patients (According to AKI and non-AKI cohorts)

| Variables | Overall | non-AKI | AKI | p-value*^2^* |
| --- | --- | --- | --- | --- |
| Number of patients | N = 1,552*^1^* | N = 1,202*^1^* | N = 350*^1^* |  |
| In-hospital mortality | 75 (4.8%) | 13 (1.1%) | 62 (18%) | <0.001*** |
| Length of Stay (days) | 6 [4-12] | 5 [3-8] | 16 [8-26] | <0.001*** |
| Follow-up Duration for AKI (days) | 4.0 [2.2-7.0] | 4.9 [3.1-7.9] | 1.1 [0.5-2.6] | <0.001*** |
| Gender |  |  |  | 0.027* |
| Female | 737 (47%) | 589 (49%) | 148 (42%) |  |
| Male | 815 (53%) | 613 (51%) | 202 (58%) |  |
| Age (years) | |  |  | 0.1 |
| < 40 years | 247 (16%) | 197 (16%) | 50 (14%) |  |
| 40-60 years | 650 (42%) | 515 (43%) | 135 (39%) |  |
| > 60 years | 655 (42%) | 490 (41%) | 165 (47%) |  |
| Race |  |  |  | <0.001*** |
| Asian | 51 (3.3%) | 41 (3.4%) | 10 (2.9%) |  |
| Black | 154 (9.9%) | 127 (11%) | 27 (7.7%) |  |
| Hispanic | 72 (4.6%) | 64 (5.3%) | 8 (2.3%) |  |
| White | 1,085 (70%) | 868 (72%) | 217 (62%) |  |
| Other | 190 (12%) | 102 (8.5%) | 88 (25%) |  |
| Marital Status | |  |  | <0.001*** |
| Divorced | 119 (7.7%) | 98 (8.2%) | 21 (6.0%) |  |
| Married | 701 (45%) | 553 (46%) | 148 (42%) |  |
| Single | 497 (32%) | 393 (33%) | 104 (30%) |  |
| Widowed | 144 (9.3%) | 112 (9.3%) | 32 (9.1%) |  |
| Other | 91 (5.9%) | 46 (3.8%) | 45 (13%) |  |
| BMI (kg/m^2^) |  |  |  | <0.001*** |
| < 30 | 689 (44%) | 568 (47%) | 121 (35%) |  |
| >=30 | 311 (20%) | 252 (21%) | 59 (17%) |  |
| Missing | 552 (36%) | 382 (32%) | 170 (49%) |  |
| SOFA score | 5.0 [3.0-8.0] | 3.0 [2.0-6.0] | 6.0 [3.0-9.0] | <0.001*** |
| SIRS score | 3.00 [2.00-4.00] | 3.00 [2.00-3.00] | 3.00 [3.00-4.00] | <0.001*** |
| Vital Signs |  |  |  |  |
| RR (insp/min) | 20 [17-24] | 20 [16-24] | 20 [17-24] | 0.6 |
| HR (bpm) | 99 [84-115] | 98 [83-114] | 99 [84-115] | 0.6 |
| SBP (mmHg) |  |  |  | <0.001*** |
| < 90 | 7 (0.5%) | 6 (0.5%) | 1 (0.3%) |  |
| 90-140 | 731 (47%) | 590 (49%) | 141 (40%) |  |
| >140 | 203 (13%) | 167 (14%) | 36 (10%) |  |
| Missing | 611 (39%) | 439 (37%) | 172 (49%) |  |
| DBP (mmHg) |  |  |  | <0.001*** |
| < 60 | 99 (6.4%) | 79 (6.6%) | 20 (5.7%) |  |
| 60-90 | 765 (49%) | 625 (52%) | 140 (40%) |  |
| >90 | 77 (5.0%) | 59 (4.9%) | 18 (5.1%) |  |
| Missing | 611 (39%) | 439 (37%) | 172 (49%) |  |
| Laboratory tests |  |  |  |  |
| RBC (m/uL) | 3.79 [3.36-4.25] | 3.82 [3.41-4.25] | 3.72 [3.19-4.34] | 0.055 |
| Hb (g/dL) | 11.57 ± 2.03 | 11.59 ± 1.90 | 11.50 ± 2.43 | 0.15 |
| WBC (K/uL) | 9.0 [6.2-13.1] | 8.3 [5.9-12.0] | 12.3 [8.2-18.2] | <0.001*** |
| PLT (K/uL) | 208 [147-275] | 211 [151-277] | 192 [133-260] | 0.002** |
| pO₂ (mmHg) |  |  |  | <0.001*** |
| < 80 | 196 (13%) | 87 (7.2%) | 109 (31%) |  |
| >=80 | 201 (13%) | 66 (5.5%) | 135 (39%) |  |
| Missing | 1,155 (74%) | 1,049 (87%) | 106 (30%) |  |
| pCO₂ (mmHg) |  |  |  | <0.001*** |
| < 35 | 122 (7.9%) | 53 (4.4%) | 69 (20%) |  |
| 35-45 | 183 (12%) | 74 (6.2%) | 109 (31%) |  |
| >45 | 92 (5.9%) | 26 (2.2%) | 66 (19%) |  |
| Missing | 1,155 (74%) | 1,049 (87%) | 106 (30%) |  |
| PH |  |  |  | <0.001*** |
| < 7.35 | 172 (11%) | 61 (5.1%) | 111 (32%) |  |
| 7.35-7.45 | 214 (14%) | 93 (7.7%) | 121 (35%) |  |
| >7.45 | 55 (3.5%) | 27 (2.2%) | 28 (8.0%) |  |
| Missing | 1,111 (72%) | 1,021 (85%) | 90 (26%) |  |
| HCO₃⁻ (mEq/L) | 24.0 [21.0-26.0] | 24.0 [22.0-27.0] | 21.5 [18.0-25.0] | <0.001*** |
| Na (mEq/L) | 139.0 [136.0-141.0] | 139.0 [136.0-141.0] | 139.0 [136.0-142.0] | 0.091 |
| K (mEq/L) | 3.90 [3.60-4.30] | 3.90 [3.60-4.20] | 4.10 [3.60-4.40] | <0.001*** |
| Ca (mg/dL) | 8.50 [7.90-8.90] | 8.50 [8.10-8.90] | 8.10 [7.40-8.70] | <0.001*** |
| Mg (mg/dL) | 1.88 [1.70-2.10] | 1.80 [1.70-2.00] | 1.90 [1.70-2.10] | 0.005** |
| Lactate (mmol/L) |  |  |  | <0.001*** |
| < 2.0 | 355 (23%) | 205 (17%) | 150 (43%) |  |
| 2.0–4.0 | 140 (9.0%) | 73 (6.1%) | 67 (19%) |  |
| > 4.0 | 47 (3.0%) | 6 (0.5%) | 41 (12%) |  |
| Missing | 1,010 (65%) | 918 (76%) | 92 (26%) |  |
| Glu (mg/dL) | 107 [89-140] | 103 [87-130] | 126 [99-175] | <0.001*** |
| TG (mg/dL) |  |  |  | <0.001*** |
| < 150 | 326 (21%) | 268 (22%) | 58 (17%) |  |
| 150-199 | 59 (3.8%) | 42 (3.5%) | 17 (4.9%) |  |
| 200-499 | 83 (5.3%) | 57 (4.7%) | 26 (7.4%) |  |
| >=500 | 51 (3.3%) | 27 (2.2%) | 24 (6.9%) |  |
| Missing | 1,033 (67%) | 808 (67%) | 225 (64%) |  |
| ALT (IU/L) | 52 [22-167] | 53 [22-172] | 48 [25-146] | 0.7 |
| AST (IU/L) | 59 [26-142] | 54 [24-135] | 73 [34-175] | <0.001*** |
| TBIL (mg/dL) | 0.90 [0.50-2.50] | 0.90 [0.50-2.20] | 1.10 [0.60-2.90] | <0.001*** |
| SCr (mg/dL) | 0.80 [0.60-1.20] | 0.80 [0.60-1.00] | 1.10 [0.70-1.90] | <0.001*** |
| UCr (mg/dL) | 13 [9-23] | 12 [8-19] | 22 [13-38] | <0.001*** |
| BUN (mg/dL) | 13 [9-23] | 12 [8-19] | 22 [13-38] | <0.001*** |
| AG (mEq/L) | 14.0 [12.0-16.0] | 14.0 [12.0-16.0] | 15.0 [13.0-18.0] | <0.001*** |
| Alb (g/dL) | 3.30 [2.90-3.70] | 3.40 [3.00-3.80] | 3.00 [2.50-3.50] | <0.001*** |
| ACAG | 16.8 [14.6-19.3] | 16.3 [14.3-18.5] | 18.8 [16.3-21.8] | <0.001*** |
| ACAG (IQR) |  |  |  | <0.001*** |
| Q1 | 388 (25%) | 352 (29%) | 36 (10%) |  |
| Q2 | 405 (26%) | 337 (28%) | 68 (19%) |  |
| Q3 | 394 (25%) | 295 (25%) | 99 (28%) |  |
| Q4 | 365 (24%) | 218 (18%) | 147 (42%) |  |
| Comorbidities |  |  |  |  |
| Hypertension | 667 (43%) | 509 (42%) | 158 (45%) | 0.4 |
| DM | 382 (25%) | 279 (23%) | 103 (29%) | 0.017* |
| Hyperlipidemia | 521 (34%) | 383 (32%) | 138 (39%) | 0.008** |
| CHD | 238 (15%) | 163 (14%) | 75 (21%) | <0.001*** |
| HF | 169 (11%) | 99 (8.2%) | 70 (20%) | <0.001*** |
| AF | 58 (3.7%) | 14 (1.2%) | 44 (13%) | <0.001*** |
| COPD | 114 (7.3%) | 74 (6.2%) | 40 (11%) | <0.001*** |
| Liver Cirrhosis | 131 (8.4%) | 90 (7.5%) | 41 (12%) | 0.012* |
| CP | 216 (14%) | 192 (16%) | 24 (6.9%) | <0.001*** |
| PC and PPC | 154 (9.9%) | 107 (8.9%) | 47 (13%) | 0.013* |
| CKD | 191 (12%) | 125 (10%) | 66 (19%) | <0.001*** |
| Sepsis | 223 (14%) | 79 (6.6%) | 144 (41%) | <0.001*** |
| Shock | 110 (7.1%) | 13 (1.1%) | 97 (28%) | <0.001*** |
| Interventive measure |  |  |  |  |
| Albumin infusion | 38 (2.4%) | 4 (0.3%) | 34 (9.7%) | <0.001*** |
| History of Surgical Procedure |  |  |  |  |
| Resection of Gallbladder | 63 (4.1%) | 59 (4.9%) | 4 (1.1%) | 0.002** |
| Common Bile Duct Dilation | 64 (4.1%) | 51 (4.2%) | 13 (3.7%) | 0.7 |

*^1^* n (%); Median [Q1-Q3]; Mean ± SD

*^2^* *p<0.05; **p<0.01; ***p<0.001

Abbreviation: AKI (Acute Kidney Injury), BMI (Body Mass Index), SOFA (Sequential Organ Failure Assessment), SIRS (Systemic Inflammatory Response Syndrome), RR (Respiratory Rate), HR (Heart Rate), SBP (Systolic Blood Pressure), DBP (Diastolic Blood Pressure), RBC (Red Blood Cell), Hb (Hemoglobin), WBC (White Blood Cell), PLT (Platelets), TG (Triglyceride), ALT (Alanine Aminotransferase), AST (Aspartate Aminotransferase), TBIL (Total Bilirubin), SCr (Serum Creatinine), UCr (Urine Creatinine), BUN (Blood Urea Nitrogen), AG (Anion Gap), Alb (Albumin), DM (Diabetes Mellitus), CHD (Coronary Heart Disease), HF (Heart Failure), AF (Atrial Fibrillation), COPD (Chronic Obstructive Pulmonary Disease), CP (Chronic Pancreatitis), PC (Pancreatic Cyst), PPC (Pancreatic Pseudocyst), CKD (Chronic Kidney Disease), ACAG (Albumin Corrected Anion Gap)
